# Lessons Learned From an Evaluation of Serious Gaming as an Alternative to Mannequin-Based Simulation Technology: Randomized Controlled Trial

**DOI:** 10.2196/21123

**Published:** 2020-09-28

**Authors:** Natalie C Benda, Kathryn M Kellogg, Daniel J Hoffman, Rollin J Fairbanks, Tamika Auguste

**Affiliations:** 1 National Center for Human Factors in Healthcare MedStar Institute for Innovation MedStar Health Washington, DC United States; 2 Division of Health Informatics Department of Population Health Sciences Weill Cornell Medicine New York, NY United States; 3 Emergency Medicine Georgetown University School of Medicine Washington, DC United States; 4 Quality and Safety MedStar Health Columbia, MD United States; 5 Women’s and Infants’ Services MedStar Washington Hospital Center Washington, DC United States; 6 Obstetrics and Gynecology Georgetown University School of Medicine Washington, DC United States; 7 Simulation Training & Education Lab (SiTEL) MedStar Health Washington, DC United States

**Keywords:** simulation training, continuing medical education, obstetrics

## Abstract

**Background:**

The use of new technology like virtual reality, e-learning, and serious gaming can offer novel, more accessible options that have been demonstrated to improve learning outcomes.

**Objective:**

The aim of this study was to compare the educational effectiveness of serious game–based simulation training to traditional mannequin-based simulation training and to determine the perceptions of physicians and nurses. We used an obstetric use case, namely electronic fetal monitoring interpretation and decision making, for our assessment.

**Methods:**

This study utilized a mixed methods approach to evaluate the effectiveness of the new, serious game–based training method and assess participants’ perceptions of the training. Participants were randomized to traditional simulation training in a center with mannequins or serious game training. They then participated in an obstetrical in-situ simulation scenario to assess their learning. Participants also completed a posttraining perceptions questionnaire.

**Results:**

The primary outcome measure for this study was the participants’ performance in an in-situ mannequin-based simulation scenario, which occurred posttraining following a washout period. No significant statistical differences were detected between the mannequin-based and serious game–based groups in overall performance, although the study was not sufficiently powered to conclude noninferiority. The survey questions were tested for significant differences in participant perceptions of the educational method, but none were found. Qualitative participant feedback revealed important areas for improvement, with a focus on game realism.

**Conclusions:**

The serious game training tool developed has potential utility in providing education to those without access to large simulation centers; however, further validation is needed to demonstrate if this tool is as effective as mannequin-based simulation.

## Introduction

**Figure 1:**
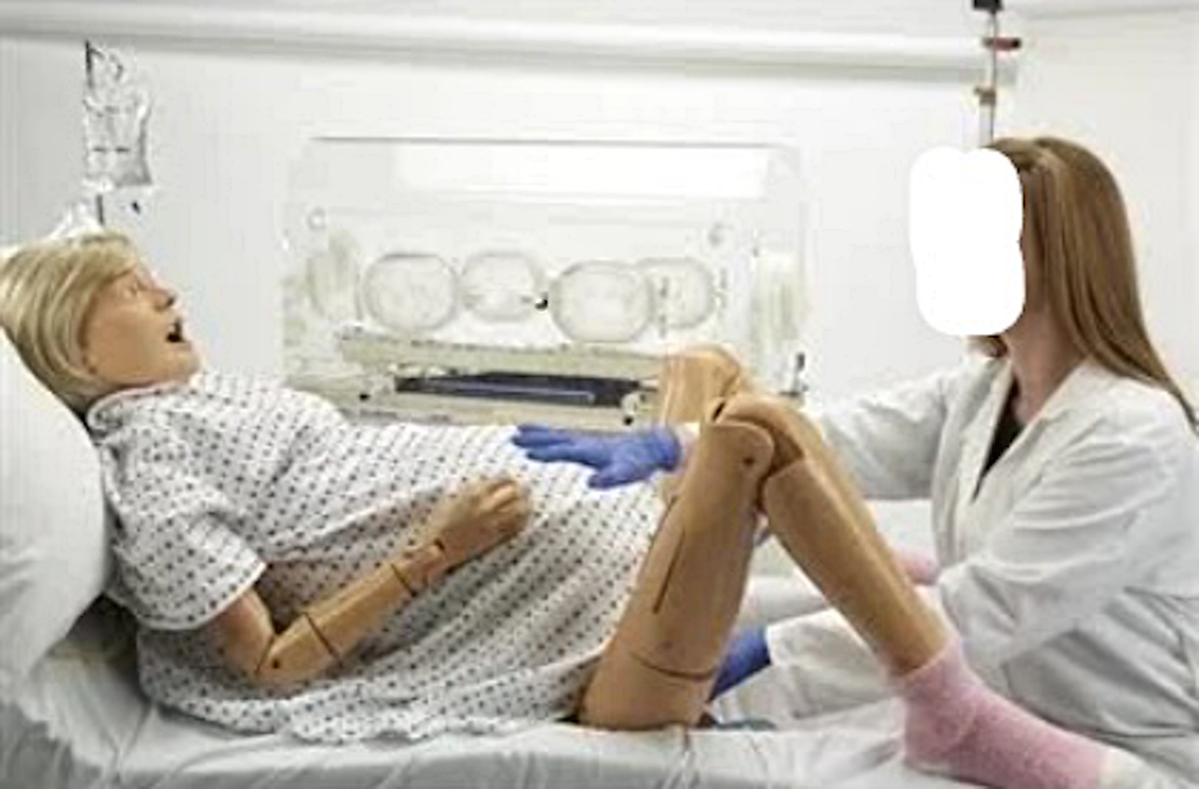
NOELLE S550 Maternal and Neonatal Birthing Simulator depiction.

Continuing education and maintenance of competency of health care professionals are critical to patient safety [1,2]. Clinical simulation training has been proven to teach and refine skills, offering the realism of the clinical environment without risks to patient safety [1,2]. However, many barriers exist to the universal availability of simulation, and an alternative simulation technology is needed. The current standard in simulation training is mannequin-based, human-patient simulators (example in Figure 1). Although its effective use has expanded rapidly in the last decade, this training modality requires expensive equipment, specialized instructors, and ongoing infrastructure support [3-5]. Widespread implementation throughout the United States has been impeded by the high cost and shortage of simulation training expertise outside of academic and large multihospital medical centers. Even in areas that do have simulation centers, few practicing physicians participate regularly [3,6].

Serious games offer a viable alternative to mannequin-based simulation. Serious games provide a learner-oriented approach, in which the learner can control the entire learning process [7,8]. This approach has also been demonstrated to have lower overall costs than mannequin-based simulation programs [8]. The use of serious games has shown promise for effectiveness in learning outcomes as compared to conventional training methods (eg, in-person or online lectures, didactic case study discussion) related to, for example, diabetes care (with primary care providers), surgical training, and emergency airway management [9-14]. However, few studies have evaluated serious games as an alternative to mannequin-based, human-patient simulators [15]. In 4 recent review articles regarding the efficacy of serious games for training health care professionals, only 1 article compared serious games to mannequin-based simulation [16-19]. Cendan and Johnson [15] compared the use of serious game and mannequin-based simulation for teaching shock physiology to second-year medical students. The authors did not detect significant differences between the serious game and mannequin-based simulation conditions in knowledge related to cardiac shock physiology and treatment among medical students. However, this study was not sufficiently powered to conclude noninferiority of the 2 treatments, and students significantly preferred mannequin-based simulation [15].

None of the 4 aforementioned review articles included serious game applications for obstetrics [16-19]. Obstetrics is one of the highest risk areas in health care [20], making it an ideal area for trialing new, innovative training techniques. Three-quarters of US obstetrician gynecologists (OBGYNs) will face a litigation claim by the age of 45 years [20]. Electronic fetal monitoring (EFM) is considered the standard of care for OBGYNs to monitor the status of the fetus during labor. Skills in EFM interpretation (determining baseline heart rate, accelerations in heart rate, decelerations in heart rate, and how this represents the current status of the fetus) and the knowledge of how to apply validated treatment protocols are critical to safe deliveries. Fetal monitoring skills are highly variable among practitioners and are difficult to teach, but clinical (mannequin-based) simulation has been proven to impart critical skills that improve patient outcomes by reducing errors and delays in care [21-25]. The importance of teaching EFM skills, coupled with the difficulties in providing widespread, accessible mannequin-based simulation to obstetrics providers and nurses, make this an excellent application area for testing the viability of serious games.

In order to address this important problem, our team of experts in education, clinical simulation, human factors engineering, obstetrics, and serious game development collaborated to create a serious game–based simulation for obstetrical training.

The aim of this study was to compare the educational effectiveness of serious game–based simulation training to traditional mannequin-based simulation training and to determine the perceptions of providers and nurses in their experience using serious game–based simulation. Our hypothesis was that the serious game–based training would be noninferior to the mannequin-based training in terms of educational effectiveness. We used EFM interpretation and decision-making skills as our test case. We also assessed participant perceptions through both quantitative and qualitative feedback to provide actionable results.

## Methods

### Setting and Population

This study was conducted across 7 diverse hospitals with obstetrical services within a not-for-profit health care system in the mid-Atlantic region of the United States. The hospitals included academic medical centers and community, urban, and suburban hospitals of varying size. The health care system averages approximately 12,000 deliveries a year.

The participants recruited for this study were attending and resident OBGYNs, midwives, and perinatal nurses. All recruited participants worked in the health care system’s labor and deliveries or mother baby units between July 2012 and November 2015. Those that had participated in beta testing of the serious game or those who would not be able to complete both phases of the study were excluded.

### Study Design

**Figure 2:**
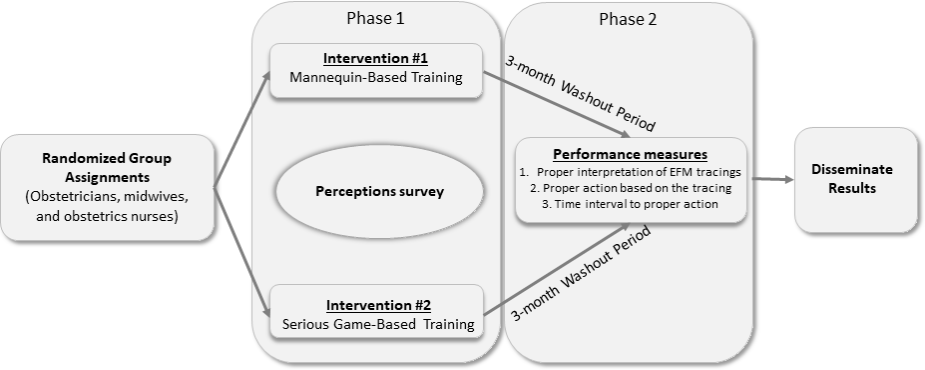
Study design flow diagram. EFM: electronic fetal monitoring.

This study utilized a mixed methods approach to evaluate the effectiveness of the new, serious game–based training method and to assess participants’ perceptions of the training. A randomized controlled trial was performed to compare the educational effectiveness of the serious game–based virtual simulation to traditional mannequin-based simulation. This study was conducted in 2 phases. In Phase 1, participants completed a verbal informed consent process and were randomized into the mannequin-based training group (Mannequin Group) or the serious game–based training group (Game Group). Upon completion of the Phase 1 training session, participants completed a questionnaire to assess their perceptions of the training. Following at least a 3-month washout period, both treatment groups participated in a posttest performance assessment. A 3-month washout period was chosen based on availability of the participants, availability of the simulation center, and the already scheduled in-situ drills that were the posttest performance assessments. This design is depicted in Figure 2. The health system’s institutional review board approved this study.

### Simulation Scenarios and Interventions

#### Simulation Scenarios

The scenarios used in both treatment groups were developed through collaboration between the subject matter experts in obstetrics and Certified Healthcare Simulation Educators at the Simulation Training & Education Lab of MedStar Health. Scenarios were designed to be representative of true clinical scenarios with clear decision-making points. They were based on simulation scenarios previously developed and used by our group for postgraduate education, postgraduate remediation, and in-situ team training drills. In each intervention group, the participant was expected to manage the simulated laboring patient in a realistic clinical scenario from triage to delivery. Time elapsed to cover 4 discrete scenarios that involved interpreting the EFM strips that were correlated to the patient’s worsening preeclampsia and fetal distress. The scenarios were delivered through 2 different interventions or mediums (described in the following sections): mannequin-based simulation training (Mannequin Group) or serious game–based simulation training (Game Group).

#### Mannequin-Based Training

Participants randomized to the Mannequin Group completed the scenario at a Simulation Training and Education Lab simulation center site involving a pregnant mannequin patient (NOELLE S550 Maternal and Neonatal Birthing Simulator, Gaumard, Miami, FL; Figure 1) with preeclampsia with severe features. Participants interacted with the mannequin as they would with a typical patient. The training was scripted into 4 discrete scenes of the scenario with a single participant being run at a time. The facilitator was 1 of 2 experienced obstetricians with at least 7 years of facilitating training for residents in simulation labs with interdisciplinary teams in in-situ drills. Pilot testing of the completed scenarios and simulation was done with a group of physicians and nurses. The training was done once, and no repetitions were permitted.

#### Serious Game–Based Training

Participants randomized to the Game Group completed the virtual simulation session on their personal computer. This simulation program runs on a standard personal computer; thus, it can be easily used in the hospital, at home, or anywhere a computer and internet connection are available. The EFM serious game was developed utilizing the MedStar Digital Simulation Platform and the Unity3D game engine. The game also went through extensive pilot and usability testing after development, with those potential learners being excluded from participating in the subsequent study. The feedback that was given in the pilot testing was used to improve the final serious game product. Most learners accessed the EFM Trainer via a personal computer using web browsers via a learning management system. Participants completed the training at their leisure; therefore, any external stimuli varied. The training was done once, and no repetitions were permitted.

**Figure 3:**
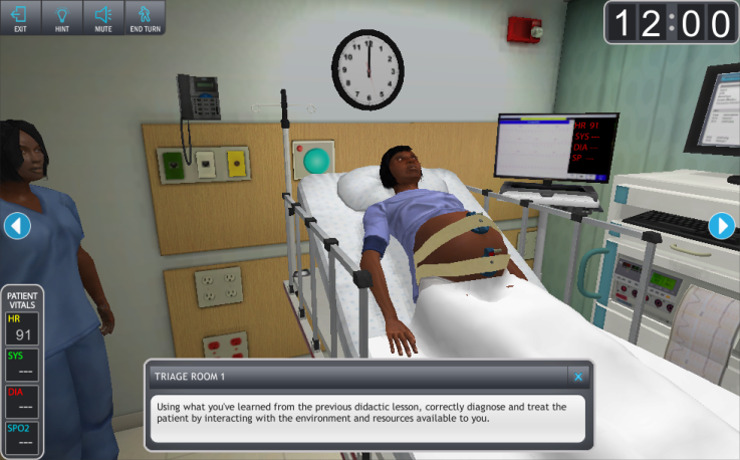
Depiction of the avatar participants utilized to navigate the serious game.

The “avatar” in the serious game represented the participant (the provider or nurse using the system). The participant’s avatar performed real-life tasks in a realistic patient scenario. This is depicted in Figure 3. The scenarios in the serious game were clinically the same as the scenarios used in the Mannequin Group. Any difference noted was slight and due to any difficulty representing the nuance in a serious game. Items like presenting symptoms, differential diagnoses, exams, laboratory values, and EFM tracings were the same. Names of patients and visual representation of the patients differed only slightly.

### Measurements

#### Posttest Performance Assessment

The Phase 2 posttest assessment involved participation in the MedStar Obstetrical Safety Training (MOST) program, a mannequin-based simulation program that served as the gold standard for performance assessment. The hospital system implemented the MOST program 10 years ago, and all practicing obstetrics physicians and nurses are required to complete it. The MOST program is a single-session, in-situ, mannequin-based simulation training program that allows perinatal teams to practice and assess various aspects of emergent obstetrical care, including interpretation and decision making related to EFM strips. For the purposes of this study, the MOST program session was used as the postintervention tool to assess the learner’s knowledge after the minimum 3-month washout period following the initial trainings described (Game Group or Mannequin Group), not as an educational program.

A newly developed evaluation tool was incorporated into the MOST training and was used to assess performance on standardized scenarios involving the need for EFM interpretation and related decision making. Using the MOST program for the evaluation was chosen because it closely approximates real-life performance. To avoid any scenario confounds, the specific scenarios selected for MOST testing were different from those presented to either group during training. The slight differences were based around the types of categories of EFM tracings and the time it took for abnormal tracings to develop.

#### Posttraining Perceptions Questionnaire

We developed a survey instrument in conjunction with a PhD-level biostatistician to assess perceptions of the intended user groups, administered at completion of Phase 1. Prior to the randomized controlled trial, the survey was pilot tested by those participating in the pilot testing for the serious game to ensure comprehensibility. The versions of the survey administered to each group differed only in wording to be relevant to the arm of the study (ie, either referred to serious game–based simulation or mannequin-based simulation). The questions regarding the participants’ experience consisted of 19 statements pertaining to the participants’ experience with technology, views on simulation, and their specific experience with this study. The participants rated these statements on a 5-point Likert scale with responses ranging from strongly disagree to strongly agree. At the conclusion of the questionnaire, participants also provided free-text narratives regarding their experience.

### Study Protocol and Data Collection

#### Prestudy: Recruitment and Randomization

Recruitment occurred via email, dissemination of paper fliers, principle investigator attendance of staff meetings, and word-of-mouth. Interested parties contacted research personnel (NB or DH) who ensured prospective participants met the inclusion criteria and obtained verbal consent. Eligible participants were then randomized to the Mannequin Group or Game Group. A randomized permuted block design of mixed block size was used to assign participants to 1 of the 2 study groups with a 1:1 ratio. The randomization sequence was generated by a PhD-level biostatistician, and allocation was performed by a trained study research assistant.

#### Phase 1: Simulation Training

Participants randomized to the mannequin-based training were first briefed by a clinical simulation specialist regarding the functionality of the mannequin and how to verbalize their interpretation of the EFM strip and subsequent actions. There was also a visible, written sign reminding the participants which components of their EFM strip interpretation needed to be verbalized.

Participants were scored based on their verbalized interpretation of the EFM strip and description of subsequent actions. Experts in simulation and obstetrics (TA and SP) used a standardized scoring sheet to score the participants’ interpretation and management. The scoring sheet was an objective tool that recorded what the participants verbalized; it has been used institutionally to create a mechanism to grade learners on their education. The observers recorded the category of tracing stated by the participant at each interpretation point. Then, the percent correct was calculated. At the end of each experience, the in-lab participants received a one-on-one debrief from the evaluator/facilitator on their interpretation of the EFM tracings and their overall decision making. The expert facilitators used the Plus/Delta debriefing model [26]. Participants were asked to identify what they felt they did well and what they would do differently if they participated in the scenario again. There was then a discussion around this, and the facilitator had an opportunity to add insight into items that they noticed were not mentioned. At the end of the debriefing sessions, participants were asked to identify several “take-aways” from the session. They were asked to think about how they will change their practice as a result of participating in this experiential activity.

Immediately following the training, participants completed a questionnaire using a computer provided in the simulation center. The questionnaire consisted of demographic information, questions regarding their experience with the simulation, and the opportunity to provide a free-text narrative of their experience.

Demographic information was collected, including employment position, years of experience, prior training experience, gender, and age. There were also Likert scale–based questions related to the participant’s experience with different technologies, their perceptions of simulation-based training modalities, as well as their perceptions regarding the simulation training they had just completed. In addition, participants could provide free-text narratives regarding their experience.

**Figure 4:**
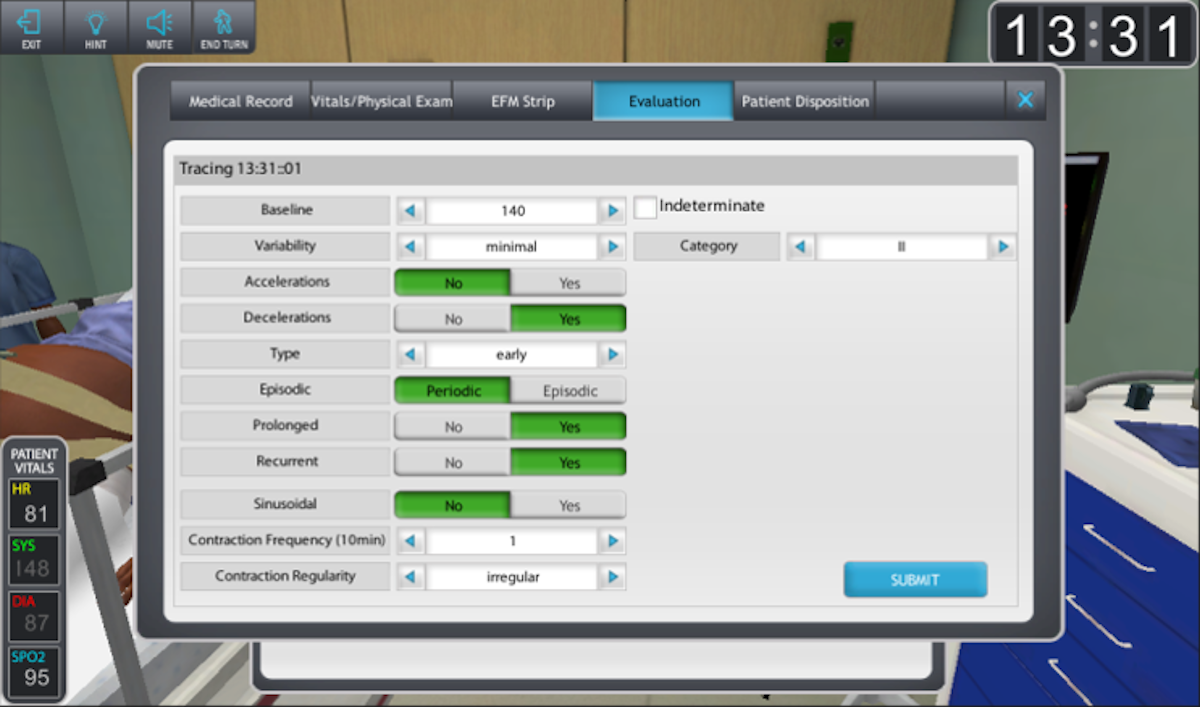
Depiction of the screen participants utilized to input answers in the serious game.

Participants randomized to the Game Group were shown a video describing the functionality of the game before they could proceed to the training scenarios. The participant used their avatar to move from room to room to manage the patient. Participants utilized the game’s controls to provide interpretations of the EFM strips and complete related patient care actions (example in Figure 4).

The game had an internal scoring mechanism equivalent to the in-lab scoring sheets. In the serious game, feedback was given based on interpretations of the fetal tracings as well as correct and incorrect actions completed by the learner. Incorrect actions triggered textual descriptions of feedback that pertained specifically to the incorrect action committed and mimicked the standardized feedback provided in the mannequin-based training.

Serious game participants completed a perceptions questionnaire identical to that completed by the mannequin-based training participants. Similarly, the questionnaire was completed on a screen to which they were directed immediately following their training.

#### Phase 2: Posttest Simulation Assessment

After participants completed Phase 1, there was a 3-6–month washout period before participants were assessed in Phase 2. To assess the EFM skills imparted to learners in both the Mannequin and Game Groups, participants were evaluated in an in-situ simulation scenario (described in previous sections) involving EFM interpretation and decision making. The scenarios for the in-situ simulation-based team training mirrored that of the mannequin-based training simulation. The scenarios contained different details from Phase 1 but were similar in the clinical topic and category of EFM tracings to evaluate as in the mannequin and serious game scenarios. Participants were first given instructions regarding the functionality of the mannequin and reminded to verbalize their EFM strip interpretations and care actions. Participants’ verbalized answers were scored by a grader (the same graders used in the mannequin-based training scenarios) in real-time. The grader stood behind an opaque screen so they could not see the participant in an effort to blind the grader from the participant’s allocation group. Correct answers to questions had been objectively determined in advance to reduce potential grader bias. Similar to Phase 1, subject matter experts and doctoral professionals with interest in assessments developed and scored the evaluations for the in-situ simulation of Phase 2.

### Data Analysis

#### A Priori Power Analysis

Prior to beginning data collection, sample size calculations were conducted based on a noninferiority design (ie, Mannequin Group was not worse than the Game Group within a predetermined margin). The noninferiority margin was set to a –5-point difference in the primary outcome measure, namely performance on the MOST training. This analysis indicated that 64 participants per group (128 total) would be required to conclude noninferiority of the mannequin-based training.

#### Quantitative Data Analysis

Following numerous different recruitment efforts using various strategies, a total of 36 participants volunteered to be part of the study. While this sample size was not sufficient for testing noninferiority of the Mannequin Group, statistical analysis was performed to compare the performance of Mannequin Group participants to that of the Game Group participants on the posttest assessment simulation to detect if there were any significant differences that may be pertinent to understanding the results or to future studies. Statistical analysis was also utilized to compare differences in perceptions from the Likert scale questions in the posttraining assessment questionnaire. Descriptive statistics were performed to calculate mean, median, and SD of the performance and demographic data collected. Comparisons of continuous variables were conducted using student *t* tests or Wilcoxon rank sum tests for skewed data. Categorical variables were compared using a chi square test or Fisher's exact test for small cell size (n<5). For statistical analysis, SAS 9.4 was utilized, and all analyses were carried out by a Masters-level biostatistician.

#### Qualitative Data Analysis

Qualitative analysis of the free-text narratives provided by participants during the posttraining questionnaire in Phase 1 was conducted utilizing thematic analysis [27]. Two researchers, one PhD-level, with extensive experience analyzing qualitative data (DH and NB) inductively elicited themes from the dataset. Inductive coding involves compiling themes as they emerge through the text, as opposed to deductive coding, which utilizes a previously developed set of themes [28]. A researcher with expertise in obstetrics (TA) then reviewed the themes and definitions for clinical accuracy and relevance. Once the themes had undergone subject matter expert review (TA), the two researchers (DH and NB) established interrater reliability with a Fleiss kappa value of 0.791, denoting substantial agreement [29]. The researchers then independently completed the coding. The completed coding was reviewed by both researchers to ensure consistency of coding. Finally, axial coding was utilized to collapse or combine similar themes [30].

## Results

### Characteristics of Study Participants

There were 36 total participants in the study. Table 1 provides demographic summary data for both participant groups. No significant differences were found in any of the demographic categories between the 2 treatment groups.

**Table 1:** Demographics of participants in the simulation (Mannequin Group) and serious game (Game Group) arms of the randomized controlled trial.

Characteristics		Game Group (n=18)	Mannequin Group (n=18)	*P* value
**Participant role, n (%)**				
	Nurse	2 (11)	2 (11)	1.00
	Resident physician	12 (67)	12 (67)	
	Attending physician	4 (22)	4 (22)	
Gender (female), n (%)		16 (89)	14 (78)	.66
Age (years), mean (SD)		32.91 (7.35)	32.74 (7.4)	.41
Years in role, mean (SD)		2.83 (3.29)	2.32 (2.41)	.99

### Quantitative Results

#### Posttest Performance Assessment

No significant differences were detected between the Mannequin and Game Groups in overall performance during Phase 2, namely the MOST scenario (measured as the percent correct; *P*=.43). Those in the Mannequin Group had an average score of 64.2%, while those in the Game Group had an average score of 53.8%.

Table 2 provides a breakdown of performance by EFM component. The Mannequin Group verbalized the correct contraction frequency more often (*P*=.04), and the Game Group was more likely to fail to state the contraction frequency (*P*=.04). None of the other tests demonstrated significant differences between groups.

**Table 2:** Performance in Phase 2 by electronic fetal monitoring (EFM) component, shown as the number and percent of participants providing the correct responses.

EFM component	Game Group (n=18), n (%)	Mannequin Group (n=18), n (%)	*P* value
Fetal heart rate category	14 (77.8)	9 (50.0)	.09
Fetal heart rate variability	6 (33.3)	9 (50.0)	.31
Declarations	15 (83.3)	16 (88.9)	1.00
Type	8 (44.4)	9 (50.0)	.74
Baseline fetal heart rate	8 (44.4)	8 (44.4)	1.00
Accelerations	11 (61.1)	11 (61.1)	1.00
Contraction frequency	5 (27.8)	11 (61.1)	.04
Recurrent	7 (40.0)	18 (100)	.06
Regularity	13 (72.2)	13 (72.2)	1.00

#### Post-Training Perceptions Questionnaire

Table 3 provides a comparison of the 2 participant groups for the 5 survey questions that pertained specifically to the participants’ experience during their training intervention. There were no significant differences in perceptions between groups.

**Table 3:** Simulation training perception survey results, reported as the average Likert scale responses on scale of 1 (strongly disagree) to 5 (strongly agree).

Survey prompt	Game Group, mean	Mannequin Group, mean	*P* value
Learning through simulation as I just experienced is frustrating.	3.11	2.33	.19
I think the simulation training I just experienced is a great tool for learning.	4.11	4.50	.46
I would like to be involved in more simulation training of the sort I just experienced.	4.00	4.11	.16
I do not see how the kind of training I just experienced is relevant to my work.	2.00	1.47	.37
I am satisfied with this training experience.	3.83	4.06	.82

### Qualitative Results

#### Summary

Of the 36 total participants, 16 (44%) voluntarily provided free-text narratives during the survey portion of Phase 1 of the study to describe their experience. From the participants’ descriptions, 6 unique themes emerged. We describe each theme in the following sections, including participant quotes, followed by a participant ID displayed such that the first letter signals the participant role (A: attending physician, R: resident physician, N: nurse), followed by an ID number, with the last letter denoting the treatment group (M: Mannequin Group, G: Game Group). Themes are listed in descending order from most to least commonly discussed by participants.

#### Overall Experience and Opinions on Simulation

Participants from both the Mannequin and Game Groups provided feedback regarding their overall experience and opinions related to simulation, which were positive, neutral, or negative. Game Group participants, for example, noted that they would “rather do this then listen to a lecture” [R-10-G]. Another described that “this would be a useful tool once you get used to what is expected” [A-07-G].

Mannequin Group participants also provided feedback about the simulation: “I liked the concept of following a patient through her labor course” [A-09-M]. Mannequin Group participants also tended to describe their views of the simulation, which varied by participant. One stated: “I strongly believe that simulation-based training is absolutely vital to training for any and all medical procedures” [N-06-M]. Conversely, another participant described, “I find simulations frustrating for their lack of natural conclusion and the feeling of being judged” [R-23-M].

#### Realism of the Simulation

Both Mannequin and Game Group participants described shortcomings related to the realism of the simulation. One Game Group participant said “I felt like I have to keep doing things and waiting for something to happen or change though that is not what I would have done in real life” [R-05-G]. Mannequin Group participants also expressed concerns, such as “It was a bit random to be having to act like I am interacting with a patient but then randomly having to do a strip review when talking to the patient” [A-09-M].

Participants also described elements that could be adapted to improve realism, such as:

Picking a [EFM] strip most like our home institution in terms of size and markings.

R-07-G

I never push IV meds, so I am not sure why I can't just call out a medication.

A-09-M

#### Navigating the Simulation and Technology Issues

Participants, predominantly from the Game Group, highlighted issues and areas for improvement related to navigating the simulation, including:

I would have liked to have a practice scenario to figure out how to virtually assess my patient.

N-03-G

There is no back button to reverse steps made (if done in error).

R-09-G

Another Game Group participant also reported that “some of the actions I performed were not recorded in the summary, and I did not get credit for them” [R-14-G].

#### Simulation as a Learning Tool

Multiple participants discussed the value of simulation as a learning tool, although this was more commonly discussed by participants in the Mannequin Group. Feedback included:

[I] look forward to improving my virtual assessment skills and score.

N-03-G

Having gone through the motions in sim lab, when the stakes weren't as high, provides comfort and give you confidence when faced with the real scenario.

R-22-M

#### Facilitators and Challenges to Understanding Simulation Feedback

Related to learning, one Game Group participant described challenges in understanding the feedback they were given via a “score card”:

The review score card did not help me understand where my clinical decision making went wrong.

N-03-G

Alternatively, one of the Mannequin Group participants noted that “in real life, it is helpful to have on-the-spot feedback” [R-02-M].

#### Mismatch in Role and Simulation Activities

Lastly, 2 nurses, 1 from each treatment group, described that they were asked to do things that fall outside of their scope of practice:

Decision to call a c/s [caesarean section] is not in my scope of practice as an L&D [labor and delivery] RN.

N-03-G

Post-partum nurses rarely have to decide what to do with MgSo4 toxicity.

N-05-M

## Discussion

This study provides lessons to help advance the future of serious game simulation techniques through qualitative and quantitative evidence. Among participants in both groups, the difference in final correct answers was not shown to be statistically significant. The serious game training tool developed has potential utility in providing education to those without access to large simulation centers; however, further validation is needed to demonstrate if this tool is as effective as mannequin-based simulation. Additional lessons were provided through the assessment of participant perceptions. Namely, feedback suggested participants were accepting of and satisfied with the new training modality. Further, open-ended feedback elicited important themes for improvement to advance the future of serious games as a training tool.

For individual components of EFM interpretation and decision making, the only component that showed statistical significance was interpretation of the uterine contraction pattern. Those in the Mannequin Group performed better. However, those in the Game Group had a significantly higher occurrence of “no answer” (ie, they failed to verbalize an answer for this component), which was counted as an incorrect response. We hypothesize that the Mannequin Group gained experience with verbalizing the contraction pattern during their training and were therefore more comfortable with this during Phase 2. Those in the Game Group had no practice with verbalization until the posttest assessment. This finding highlights a type of experience gained from a simulation center that may be difficult to replicate in serious games and needs to be considered in the design of future studies.

Subjective responses, including those from the questionnaires and narrative feedback (see Qualitative Analysis), highlighted positives and room for improvement in both groups. Notably, in the perceptions survey, both groups stated the simulation modality they had experienced was a great tool for learning, they wanted to be involved in that simulation modality more, and they were satisfied with their training. This suggests that participants were generally accepting of the new training modality (serious game–based simulation). These findings were also reflected in the qualitative analysis themes related to “overall experience” and “simulation as learning tool.”

Qualitative feedback from the serious game group showed that some users found the game challenging to navigate and experienced technical difficulties. Suggestions to improve serious game play include providing practice scenarios, providing better affordances related to how to complete a scenario, and incorporating an “undo” feature. The Game Group did watch a mandatory video, but the finding related to practice sessions suggests interactive tutorials and some type of competency assessment prior to using the training game may be beneficial. In general, maneuvering through the game must be simple. We completed a usability study prior to the assessment reported here, but these new findings underscore the importance of user-centered design as an iterative process that occurs throughout the product development lifecycle. Designing serious gaming experiences to suit user needs and goals may encourage self-guided mastery of competencies, particularly in an area as crucial and challenging as EFM interpretation.

Participants from both groups described challenges related to realism of the simulation. One participant suggested that the serious game–based simulation may benefit from using EFM strips similar to what is used in their hospital. This highlights an important point for expanding serious game use to national and international platforms. Specifically, it suggests that it may be beneficial to offer customization settings so the game feels as realistic as possible. Nurse participants also described that some of the actions fell outside of their scope of practice. Relatively few studies have incorporated nurses into serious game development and assessment [16-19], and it is important to ensure participants of various roles have opportunities for serious game–based learning. However, our finding highlights the need for adapted scenarios that accurately match the responsibilities of different roles.

The novelty of serious games for delivery of health care education will have to endure a learning curve in learners’ comfort levels of navigation. This learning curve can be mitigated through iterative testing to ensure trainings are usable for the various end-user groups. Over time, serious games for health care delivery have the potential to become mainstream, much like mannequin-based simulation has over the past 10-15 years. In traditional simulation education, it is important that the level of fidelity or realism of the training matches the objectives of the training with regards to the patient, clinical facilities, and clinical scenario [31]. Understanding levels of realism across the patient, clinical facilities, and clinical scenarios necessary to meet objectives related in the serious game format will need to be similarly considered as this type of simulation training gains traction in the future.

### Limitations

Our study was limited by the difficulty in recruitment of participants. No statistically significant differences were detected in the primary outcome measure, but sufficient power was not attained to conclude equivalence of the original training method (mannequin-based simulation) to the serious game–based training. Further investigation is needed to determine if the methods are equivalent, but this study certainly supports the continued investigation of the value of virtual simulation environments such as serious game–based scenarios as alternatives to traditional mannequin-based simulation. While all participants had a washout period of at least 3 months, due to their schedules, some had washout periods as long as 6 months. This had an unknown effect on outcomes. Future studies may track this period as a covariate to test its potential effect on outcome measures.

The perception-based questionnaires were pilot tested but were not psychometrically validated, which should be considered in the interpretation of these results.

Another potential limitation may be the possible training effect from both methodologies. Though not a desired outcome, we recognize that there is sustained difficulty in interpretation of EFM regardless of type of training. Though years of experience may help, there is still significant difficulty in predicting fetal wellbeing in all EFM situations, particularly category 2 tracings [32,33].

The qualitative analysis was limited in that free-text narratives were not mandatory and only provided by 44% of the participants. Future studies may employ more robust techniques, such as semistructured interviews, to gain more comprehensive feedback from all participants. The discovered qualitative themes, however, still provide important insight into improving the future of serious game–based simulation.

This study was conducted specifically with OBGYN providers and nurses, which may not necessarily be generalizable to other clinical subspecialties.

### Conclusions

Data indicated that the serious game is viewed as effective both by physician and nurse participants, and quantitative measures suggest that serious game participants will have similar performance to those participating in the human-patient simulation. However, the study was not sufficiently powered to assess our hypotheses. Larger studies are necessary before definitive conclusions can be made about how serious game training compares to the more well-established mannequin-based training methods in obstetrics as well as other specialties. We have demonstrated feasibility of using serious game training to deliver education as an alternative to simulation-based training and provided insight to improve these methods in future implementations of this technology. Our study has indicated that a focus on realism and usability of the training tool will be important areas of focus in serious game development in the future.

## Abbreviations

EFM: electronic fetal monitoring
MOST: MedStar Obstetrical Safety Training
OBGYN: obstetrician gynecologist

## References

[1] American College of Obstetricians and Gynecologists Committee on Patient SafetyQuality Improvement (2014). Committee opinion no. 590: preparing for clinical emergencies in obstetrics and gynecology. Obstet Gynecol.

[2] American College of Obstetricians and Gynecologists Committee on Patient Safety and Quality Improvement (2009). ACOG Committee Opinion No. 447: Patient Safety in Obstetrics and Gynecology.

[3] Weinstock PH, Kappus LJ, Kleinman ME, Grenier B, Hickey P, Burns JP (2005). Toward a new paradigm in hospital-based pediatric education: the development of an onsite simulator program. Pediatr Crit Care Med.

[4] Weinstock PH, Kappus LJ, Garden A, Burns JP (2009). Simulation at the point of care: Reduced-cost, in situ training via a mobile cart. Pediatric Critical Care Medicine.

[5] Rodgers D (2007). High-fidelity patient simulation: a descriptive white paper report. Healthcare Simulation Strategies.

[6] Morgan PJ, Cleave-Hogg D (2002). A worldwide survey of the use of simulation in anesthesia. Can J Anesth/J Can Anesth.

[7] Maheu-Cadotte M, Cossette S, Dubé V, Fontaine G, Mailhot T, Lavoie P, Cournoyer A, Balli F, Mathieu-Dupuis G (2018). Effectiveness of serious games and impact of design elements on engagement and educational outcomes in healthcare professionals and students: a systematic review and meta-analysis protocol. BMJ Open.

[8] Ricciardi F, De Paolis LT (2014). A Comprehensive Review of Serious Games in Health Professions. International Journal of Computer Games Technology.

[9] LeFlore JL, Anderson M, Zielke MA, Nelson KA, Thomas PE, Hardee G, John LD (2012). Can a Virtual Patient Trainer Teach Student Nurses How to Save Lives—Teaching Nursing Students About Pediatric Respiratory Diseases. Simulation in Healthcare: The Journal of the Society for Simulation in Healthcare.

[10] Khanal P, Vankipuram A, Ashby A, Vankipuram M, Gupta A, Drumm-Gurnee D, Josey K, Tinker L, Smith M (2014). Collaborative virtual reality based advanced cardiac life support training simulator using virtual reality principles. J Biomed Inform.

[11] Dankbaar MEW, Richters O, Kalkman CJ, Prins G, Ten Cate OTJ, van Merrienboer JJG, Schuit SCE (2017). Comparative effectiveness of a serious game and an e-module to support patient safety knowledge and awareness. BMC Med Educ.

[12] Knight JF, Carley S, Tregunna B, Jarvis S, Smithies R, de Freitas S, Dunwell I, Mackway-Jones K (2010). Serious gaming technology in major incident triage training: a pragmatic controlled trial. Resuscitation.

[13] Graafland M, Bemelman WA, Schijven MP (2017). Game-based training improves the surgeon's situational awareness in the operation room: a randomized controlled trial. Surg Endosc.

[14] Diehl LA, Souza RM, Gordan PA, Esteves RZ, Coelho ICM (2017). InsuOnline, an Electronic Game for Medical Education on Insulin Therapy: A Randomized Controlled Trial With Primary Care Physicians. J Med Internet Res.

[15] Cendan JC, Johnson TR (2011). Enhancing learning through optimal sequencing of web-based and manikin simulators to teach shock physiology in the medical curriculum. Adv Physiol Educ.

[16] Ijaz A, Khan MY, Ali SM, Qadir J, Boulos MNK (2019). Serious games for healthcare professional training: A systematic review. European Journal of Biomedical Informatics.

[17] Sharifzadeh N, Kharrazi H, Nazari E, Tabesh H, Edalati Khodabandeh M, Heidari S, Tara M (2020). Health Education Serious Games Targeting Health Care Providers, Patients, and Public Health Users: Scoping Review. JMIR Serious Games.

[18] Chon S, Timmermann F, Dratsch T, Schuelper N, Plum P, Berlth F, Datta RR, Schramm C, Haneder S, Späth MR, Dübbers M, Kleinert J, Raupach T, Bruns C, Kleinert R (2019). Serious Games in Surgical Medical Education: A Virtual Emergency Department as a Tool for Teaching Clinical Reasoning to Medical Students. JMIR Serious Games.

[19] Gorbanev I, Agudelo-Londoño S, González RA, Cortes A, Pomares A, Delgadillo V, Yepes FJ, Muñoz Ó (2018). A systematic review of serious games in medical education: quality of evidence and pedagogical strategy. Med Educ Online.

[20] Jena AB, Seabury S, Lakdawalla D, Chandra A (2011). Malpractice Risk According to Physician Specialty. N Engl J Med.

[21] McGaghie WC, Issenberg SB, Petrusa ER, Scalese RJ (2006). Effect of practice on standardised learning outcomes in simulation-based medical education. Med Educ.

[22] Deering S, Johnston LC, Colacchio K (2011). Multidisciplinary teamwork and communication training. Semin Perinatol.

[23] Merién AR, van de Ven J, Mol B, Houterman S, Oei S (2010). Multidisciplinary Team Training in a Simulation Setting for Acute Obstetric Emergencies.

[24] Wayne DB, Butter J, Siddall VJ, Fudala MJ, Linquist LA, Feinglass J, Wade LD, McGaghie WC (2005). Simulation-based training of internal medicine residents in advanced cardiac life support protocols: a randomized trial. Teach Learn Med.

[25] Wayne DB, Didwania A, Feinglass J, Fudala MJ, Barsuk JH, McGaghie WC (2008). Simulation-based education improves quality of care during cardiac arrest team responses at an academic teaching hospital: a case-control study. Chest.

[26] Brown M, Holt R (2015). Utilizing Plus/Delta Debriefing to Enhance Learning in Phlebotomy Simulations. Am J Clin Pathol.

[27] Strauss A, Corbin J (2015). Basics of Qualitative Research: Techniques and Procedures for Developing Grounded Theory.

[28] Miles M, Huberman A, Saldana J (2014). Qualitative Data Analysis: A Methods Sourcebook.

[29] Landis JR, Koch GG (1977). The Measurement of Observer Agreement for Categorical Data. Biometrics.

[30] Saldana J (2015). The Coding Manual for Qualitative Researchers. 2nd ed.

[31] Tun JK, Alinier G, Tang J, Kneebone RL (2015). Redefining Simulation Fidelity for Healthcare Education.

[32] Shepherd E, Salam R, Middleton P, Makrides M, McIntyre S, Badawi N, Crowther CA (2017). Antenatal and intrapartum interventions for preventing cerebral palsy: an overview of Cochrane systematic reviews. Cochrane Database Syst Rev.

[33] Macones G, Hankins G, Spong C, Hauth J, Moore T (2008). The 2008 National Institute of Child Health and Human Development workshop report on electronic fetal monitoring: update on definitions, interpretation, and research guidelines. J Obstet Gynecol Neonatal Nurs.

